# RACE AND AGE DIFFERENCES IN DEPRESSION AND ANXIETY DURING COVID-19: A LONGITUDINAL STUDY

**DOI:** 10.1093/geroni/igac059.2974

**Published:** 2022-12-20

**Authors:** Xiaochuan Wang, Mengyu Xu, Kim Gryglewicz, Susanny Beltran

**Affiliations:** University of Central Florida, Orlando, Florida, United States; University of Central Florida, Orlando, Florida, United States; University of Central Florida, Orlando, Florida, United States; University of Central Florida, Orlando, Florida, United States

## Abstract

The COVID-19 pandemic has negatively affected the physical and mental health of many. It remains unclear whether such impacts differ across diverse races or age groups, or along the course of the pandemic. This study assessed trends in levels of depression and anxiety symptoms among U.S. adults during COVID-19 and whether differences emerged across race and age. Data were drawn from the Understanding America Study (UAS) COVID-19 survey, a longitudinal panel survey of a nationally representative sample of over 6,000 individuals. A mixed effect linear model was conducted to assess the influence of race and age on the level of depression and anxiety over time during the pandemic, controlling for covariates such as marital status, employment status, and household income. Results indicate that greater age was associated with lower levels of depression and anxiety. Additionally, trends in levels of depression and anxiety vary across races (e.g., minority populations generally reported lower or comparable levels of depression and anxiety comparing to the White). Findings further suggest significant interactions between age and race, especially among minoritized adults. Study findings underscore the importance of future research and tailored strategies to improve culturally sensitive and age-appropriate mental health services targeting diverse populations.

